# Molecular characterization and new genotypes of *Enterocytozoon bieneusi* in pet chipmunks (*Eutamias asiaticus*) in Sichuan province, China

**DOI:** 10.1186/s12866-018-1175-y

**Published:** 2018-04-18

**Authors:** Lei Deng, Wei Li, Zhijun Zhong, Yijun Chai, Leli Yang, Hang Zheng, Wuyou Wang, Hualin Fu, Min He, Xiangming Huang, Zhicai Zuo, Ya Wang, Suizhong Cao, Haifeng Liu, Xiaoping Ma, Kongju Wu, Guangneng Peng

**Affiliations:** 1 The Key Laboratory of Animal Disease and Human Health of Sichuan Province, College of Veterinary Medicine, Sichuan Agricultural University, Province, Chengdu, 611130 Sichuan China; 2 Chengdu Giant Panda Breeding Research Base, Province, Chengdu, 625001 Sichuan China

**Keywords:** *Enterocytozoon bieneusi*, Chipmunks, Prevalence, Genotype, Zoonotic potential

## Abstract

**Background:**

*Enterocytozoon bieneusi*, the most commonly identified microsporidian species in humans, is also identified in livestock, birds, rodents, reptiles, companion animals, even wastewater. However, there is no information available on occurrence of *E. bieneusi* in pet chipmunks. The aim of the present study was to determine the genotypes, molecular characterization of *E. bieneusi* in pet chipmunks, and assess the zoonotic potential.

**Results:**

A total of 279 fecal specimens were collected from chipmunks from seven pet shops and one breeding facility in Sichuan province, China. The prevalence for *E. bieneusi* was 17.6% (49/279) based on nested PCR targeting the internal transcribed spacer (*ITS*) region*.* The prevalence of *E. bieneusi* in chipmunks < 90 days of age was significantly higher than that in older chipmunks; however, differences among different sources and between genders were not significant. Eight genotypes of *E. bieneusi* were identified, including four known genotypes (D, Nig7, CHG9, and CHY1) and four novel genotypes (SCC-1 to 4). Phylogenetic analysis classified these genotypes into four distinct groups as follows: genotypes D and CHG9 clustered into group 1 of zoonotic potential; genotypes Nig7 and CHY1 clustered into group 6 and a new group, respectively; the four novel genotypes (SCC-1 to 4) formed a separate group named group 10.

**Conclusions:**

To the best of our knowledge, this is the first study reporting the prevalence and genotypes of *E. bieneusi* in pet chipmunks in China. Genotypes D and Nig7, found in chipmunks in this study, have also been previously identified in humans, which suggests that chipmunks might play a role in the transmission of this pathogen to humans.

## Background

Microsporidia comprise a large and diverse group of intracellular eukaryotes that infects invertebrate and vertebrate hosts worldwide; to date, it consists of approximately 1300 species in 160 genera [1]. *Enterocytozoon bieneusi* is the most frequently detected species of microsporidia and is responsible for greater than 90% of human microsporidiosis cases [2, 3]. *E. bieneusi* usually causes self-limiting diarrhea and malabsortion in healthy individuals [4]. However, it can cause life-threatening diarrhea in individuals with deficient immune systems, such as AIDS patients and transplant recipients [5].

Genotypes of *E. bieneusi* have been determined based on sequence analysis of the internal transcribed spacer (*ITS*) region of ribosomal RNA (rRNA) [1]. To date, more than 240 genotypes of *E. bieneusi* have been identified in humans and animals [6, 7]. The *ITS* genotypes of *E. bieneusi* have been divided into nine different groups based on phylogenetic analyses [8]. Group 1, considered zoonotic, is frequently identified in humans and animals [9]. In contrast, the remaining groups (groups 2–9) are considered host-adapted groups and have no significant public health importance [10, 11].

In China, *E. bieneusi* has been reported in humans, livestock, companion animals, and wastewater, and some genotypes of this species have been identified in both humans and animals [10, 12, 13]. Chipmunks (*Eutamias asiaticus*) have become popular in China as companion animals. However, there is no epidemiological information regarding the prevalence of *E. bieneusi* in chipmunks. The aim of this study was to determine the prevalence and genotypes of *E. bieneusi* in chipmunks, as well as to assess the zoonotic potential of this organism as it relates to pet chipmunks and humans.

## Method

### Collection of specimens

A total of 279 fecal specimens were obtained from chipmunks between March 2016 and April 2017 from seven pet shops and one breeding facility in Sichuan province, southwestern China (Table 1). The fecal samples were collected from the bottom of cages after defecation and then immediately placed into individual 30-mL sterile containers. All the fecal samples were taken to the laboratory in a cooler with ice packs within 24 h. All the chipmunks were in apparently good health at the time sampling and the age, gender and source was also recorded at the same time.

**Table 1:** Prevalence and genotypes of *E. bieneusi* in pet chipmunks from different sources in Southwestern China

Source	No. of animals	No. of positive (%)	Genotypes (n)
Pet shop1	24	7 (29.2%)	D (2); CHG9 (1); SCC-1 (4)
Pet shop2	30	4 (13.3%)	SCC-1 (4)
Pet shop3	28	6 (21.4%)	D (1); CHY1 (2); SCC-3 (3)
Pet shop4	14	2 (14.3%)	SCC-3 (2)
Pet shop5	19	6 (31.6%)	Nig7 (2); SCC-2 (4)
Pet shop6	35	6 (17.1%)	CHG9 (1); SCC-2 (5)
Pet shop7	26	5 (19.2%)	SCC-1 (5)
Breeding facility	103	13 (12.6%)	D (3); Nig 7 (2); CHY1 (3); SCC-1 (4); SCC-4 (1)
Total	279	49 (17.6%)	D (6); Nig 7 (4); CHG9 (2); CHY1 (5); SCC-1 (17); SCC-2 (9); SCC-3 (5); SCC-4 (1)

### DNA extraction and PCR amplification

All the fecal specimens were washed three times by centrifugation at 1500 g for 10 min with distilled water. Genomic DNA was extracted from approximately 200 mg of each processed fecal specimen using the E.Z.N.A.R® Stool DNA kit (Omega Biotek Inc., Norcross, USA) according to the manufacturer’s recommended instructions. The extracted DNA was stored at − 20 °C until molecular analysis.

*E*. *bieneusi* was determined by nested PCR amplification of a 392-bp fragment, containing the entire *ITS* (243 bp) and the portions of the flanking large and small subunits of the rRNA gene. The primers and cycling conditions in nested PCR were used as previously described by Sulaiman et al. [14]. TaKaRa Taq™ DNA Polymerase (TaKaRa Bio, Otsu, Japan) was used for PCR amplifications. A negative control with no DNA added was included in all the PCR tests. The secondary PCR products were examined by agarose gel electrophoresis and visualized after ethidium bromide staining.

### Sequence and phylogenetic analyses

All amplified products were sequenced by Life Technologies (Guangzhou, China) using a BigDye® Terminator v3.1 cycle sequencing kit on an ABI 3730 DNA Analyzer (Applied Biosystems, Foster City, CA). Nucleotide sequence accuracy was confirmed by sequencing of two separate PCR products. The obtained sequences in this study were aligned with reference sequences downloaded from GenBank using the program ClustalX 2.0 (http://www.clustal.org/) to determine the genotypes. The genotypes from this study were compared with previously published *E. bieneusi* ITS genotypes using a neighbor-joining analysis of the aligned *E. bieneusi* sequences implemented in the program Mega 6 (http://www.megasoftware.net/), and a bootstrap analysis with 1000 replicates was performed to assess the robustness of clusters.

### Statistical analysis

Differences in infection rates were compared using the chi-square test and difference was considered significant when *p <* 0.05. The analysis was done using SPSS version 17.0 (SPSS Inc., Chicago, IL USA).

## Results

### Prevalence of *E. bieneusi* in chipmunks

Of the 279 fecal samples examined for *E. bieneusi* by PCR amplification of the *ITS* gene, 49 (17.6%) were positive. All tested pet shops have *E. bieneusi* infection, and infection rates ranged from 12.6 to 31.6% (Table 1). The highest infection rate was observed in pet shop 5 (31.6%, 6/19), and it was apparently higher than that in other pet shops, but the difference was not significant (*P* > 0.05). Infection rates of *E*. *bieneusi* in chipmunks of different ages and sexes are shown in Table 2. The highest prevalence of *E. bieneusi* was observed in chipmunks < 90 days of age (24%, 35/146), followed by that in 90–270-day-old chipmunks (15.1%, 8/53), and in > 270-day-old chipmunks (7.5%, 6/80); the differences among these groups was significant (*P* < 0.05). The prevalence of *E. bieneusi* was also higher in females (19.5%) than in males (15.2%), but the difference was not significant (*P* > 0.05).

**Table 2:** Prevalence and genotypes of *E. bieneusi* in pet chipmunks by age and gender

Group	No. of animals	No. of positive	Infection rate
Age			
< 90 days	146	35	24.0%
90–270 days	53	8	15.1%
> 270 days	80	6	7.5%
Sex			
Male	125	19	15.2%
Female	154	30	19.5%

### Genotype distribution and genetic characterization of *E. bieneusi* in chipmunks

Eight genotypes were identified in the present study by sequence analysis of the *ITS* gene of 49 *E. bieneusi*-positive fecal specimens; these genotypes included four known genotypes (D, Nig7, CHG9, and CHY1) and four novel genotypes named SCC-1 to SCC-4. Among these genotypes, genotype SCC-1 was the most prevalent (34.7%, 17/49), followed by SCC-2 (18.4%, 9/49), and D (12.2%, 6/49). Five genotypes were identified in the breeding facility, including two known zoonotic genotypes, D and Nig7.

**Fig. 1:**
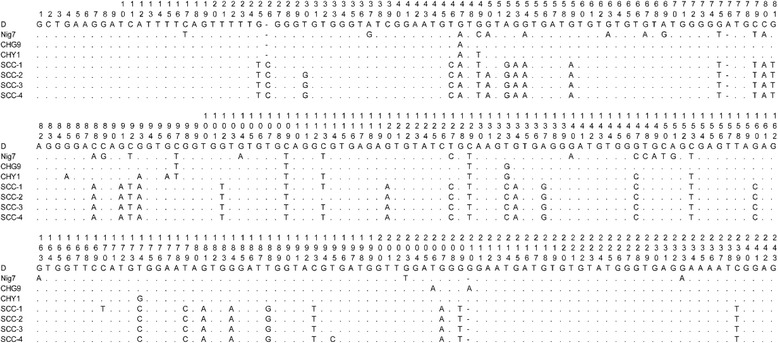
Sequence variation in the *ITS* region of the rRNA gene of *Enterocytozoon bieneusi* isolates from pet chipmunks. The *ITS* sequences of four known genotypes (D, Nig7, CHG9, and CHY1) and the four novel genotypes (SCC-1 to 4), identified in this study, were aligned with each other. The dots and transverse lines indicate base identities and deletions, respectively, relative to the *ITS* sequence of genotype D

A high degree of genetic polymorphism was observed among the novel genotypes. The base variation of the novel genotypes within the 243 bp of the *ITS* sequence is presented in Fig. 1.

### Phylogenetic analysis

**Fig. 2:**
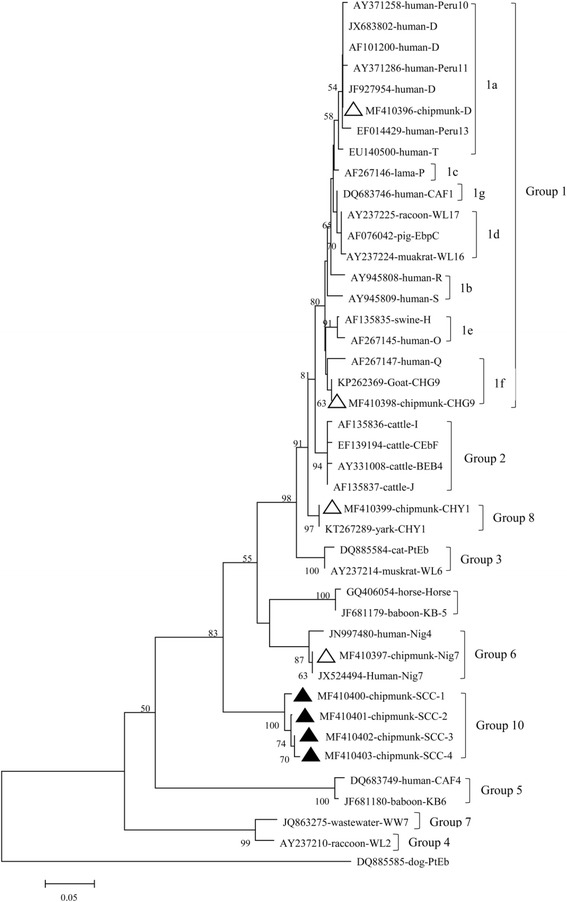
Phylogenetic relationship of *Enterocytozoon bieneusi* genotypes identified in this study and other genotypes previously deposited in GenBank as inferred by a neighbor-joining analysis of ITS sequences based on genetic distances calculated by the Kimura 2-parameter model. The number on the branches are percent bootstrapping values from 1000 replicates, with values of more than 50% shown in the tree. Each sequence is identified by its accession number, host origin, and genotype. Genotypes with *open triangles* and *black triangles* are known and novel genotypes identified in this study, respectively

Phylogenetic analysis, using the neighbor-joining method based on the *ITS* sequences of *E*. *bieneusi*, showed that all positive samples found in the present study belonged to four groups. Genotypes D and CHG9 clustered into group 1 and were further classified into subgroups 1a and 1f, respectively (Fig. 2). Genotype Nig7 clustered into group 6, and genotype CHY1 was classified as a new cluster. The four novel genotypes (SCC-1 to 4) were separated into a new group, named group 10.

## Discussion

In this study, we determined for the first time, the prevalence (17.6%) of *E. bieneusi* in chipmunks in China. At present, only two studies have described the prevalence of *E. bieneusi* in pet rodents in China; specifically, 3.6% of 140 pet chinchillas in Henan province and 16.7% of 144 pet red-bellied tree squirrels in Sichuan province were found to harbor this pathogen [6, 15]. However, there have been many reports of wild rodents with *E*. *bieneusi* infection, worldwide; a high prevalence (38.9%) was found in wild small rodents in Poland [16], 26.8% incidence was identified in wild rodents in New York [17], and 10.7% of wild mice were infected in the Czech Republic [18], whereas the lowest prevalence (1.0%) was identified in wild mice in Slovakia [19]. Differences between these studies could be explained by different geographical regions, sample sizes, management methods, age, and seasonal variations.

We also identified eight genotypes by analyzing the *ITS* sequences; these genotypes included four known genotypes (D, Nig7, CHG7, and CHY1) and four novel genotypes (SCC-1 to 4). In previous studies, eight genotypes (D, C, H, EbpA, Peru 8, S6, CZ3, and PigEBITS5) were identified in wild mice in a hybrid zone across the Czech Republic-Germany border [18], 12 (D, gorilla 1, and WR1–10) were found in wild rodents in Poland [16], two (D and BEB6) in pet chinchillas in China [15], and five (D, EbpC, SC02, CE01, and CE02) in red-bellied tree squirrels in China [6]. Together, these results show that genotype D has widespread geographical distribution and is very common in rodents. In addition, genotype D has also been identified in various hosts in China, such as humans, non-human primates, cattle, pigs, dogs, foxes, cats, goats, horses, and sheep, as well as in wastewater [5, 9, 11, 12, 20–22]. Genotype D has already been considered as a zoonotic genotype of public health significance. Genotype Nig7 was originally reported in HIV-infected patients in Nigeria [23], and genotypes CHG9 and CHY1 have been previously identified in goats and yaks in China [21, 24], respectively. These genotypes were all identified for the first time in chipmunks in China, suggesting that chipmunks play a potential role in the transmission of *E. bieneusi* to humans and other animals, acting as a reservoir host.

Genetic relationships between the *E. bieneusi* genotypes obtained in this study and known strains were identified based on phylogenetic analysis. The two known genotypes D and CHG9 belonged to group 1, which is composed of genotypes almost exclusively from humans [25–28]; this result suggested the potential for zoonotic transmission and indicates the public health significance of these genotypes [29]. Genotype CHY1 was classified as being a member of a new cluster, which contains genotypes from different animals, such as CHB1 from bears and CHK1–2 from kangaroos [10]. Genotype Nig7 clustered into group 6; this group was first identified in wastewater and has been determined to be capable of infecting a broad range of hosts including humans, non-human primates, horses, and squirrels [5, 30, 31]. The remaining four novel genotypes (SCC-1 to SCC-4) were clustered into a separate cluster, which is divergent from other known genotype groups, and appears to be specific to chipmunks; we named this Group 10. However, it remains difficult to determine if the novel genotypes have the ability to cause human microsporidiosis and have a broader host spectrum; future studies should be aimed at investigating the potential of the genotypes in these groups to cause disease in humans and other animals.

## Conclusions

This is the first report of the prevalence and genotypes of *E*. *bieneusi* in chipmunks from China; this study also identified a new chipmunk-specific group, named group 10. The detection of two known genotypes (D and Nig7) that are also common to humans and the fact that the genotype CHG9 belonged to group 1 suggests that chipmunks infected with *E. bieneusi* might pose a threat as a route of transmission to humans.

## Abbreviations

E. bieneusi: Enterocytozoon bieneusi
ITS: internal transcribed spacer
rRNA: ribosomal RNA
